# Supplementary material for: Multiplex PCR detection of enteric pathogens in a community-based birth cohort in Ecuador: comparison of xTAG-GPP and TaqMan array card assays
Source: medRxiv. 2024 Oct 18:2024.10.10.24315212. Originally published 2024 Oct 11. Preprint. [Version 2] doi: 10.1101/2024.10.10.24315212 (PMC11482970; doi:10.1101/2024.10.10.24315212)

medRxiv preprint doi: https://doi.org/10.1101/2024.10.10.24315212; this version posted October 18, 2024. The copyright holder for this preprint (which was not certified by peer review) is the author/funder, who has granted medRxiv a license to display the preprint in perpetuity.
It is made available under a CC-BY 4.0 International license .

Supplementary Information

Supplemental Table 1: Luminex Gastrointestinal Pathogen Panel (GPP) assay targets and corresponding median fluorescence intensity (MFI) thresholds for positivity

**Analyte**	**Threshold for positivity (MFI)**
Adenovirus *40/41*	≥ 150
Camplobacter	≥ 150
C. difficile Probe-1	≥ 150
C. difficile Probe-2	≥ 150
Cryptosporidium	≥ 250
E. coli O157	≥ 150
E. histolytica	≥ 250
ETEC probe-1	≥ 200
ETEC probe-2	≥ 200
Giardia	≥ 250
Norovirus Probe-1	≥ 200
Norovirus Probe-2	≥ 350
Rotavirus A	≥ 150
Salmonella Probe-1	≥ 100,000 (POS), < 300 (NEG)
Salmonella Probe-2	≥ 200
STEC Probe-1	≥ 150
STEC Probe-2	≥ 150
Shigella	≥ 150
V. cholerae	≥ 150

medRxiv preprint doi: https://doi.org/10.1101/2024.10.10.24315212; this version posted October 18, 2024. The copyright holder for this preprint (which was not certified by peer review) is the author/funder, who has granted medRxiv a license to display the preprint in perpetuity.
It is made available under a CC-BY 4.0 International license .

Supplemental Table 2: TaqMan Array Card (TAC) assay gene targets and corresponding forward (F) and reverse (R) primers and probe (P) sequences.

Organism	Gene Target	Sequence
Adenovirus (40/41)	*Fiber gene*	*F, AACTTTCTCTCTTAATAGACGCC*<br>*R, AGGGGGCTAGAAAACAAAA*<br>*P, FAM-CTGACACGGGCACTCT-MGB*
Norovirus GI	*ORF1-ORF2*	*F, CGYTGGATGCGNTTYCATGA*<br>*R, CTTAGACGCCATCATCATTYAC*<br>*P, FAM-TGGACAGGAGATCGC-MGB*
Norovirus GII	*ORF1-ORF2*	*F, CARGARBCNATGTTYAGR TGGATGAG*<br>*R, TCGACGCCATCTTCATTCACA*<br>*P, FAM-TGGGAGGGCGATCGCAATCT-MGB*
Rotavirus	*NSP3*	*F, ACCATCTWCACRTRACCCTCTATGAG*<br>*R, GGTCACATAACGCCCCTATAGC*<br>*P, FAM-AGTTAAAAGCTAACACTGTCAAA-MGB*
Camplobacter jejuni	*cadF*	*F, CWGCTAAACCATARAAATAAAATTTCTCAC*<br>*R, YTTTGAAGGTAATTTAGATATGGATAATCG*<br>*P, VIC-CATTTTGAYGATTTTTGGCTTGA-MGB*
	hipO	*F, CTTGCGGTCATGATGGACATAC*<br>*R, AGCACCACCCAAACCCTCTTCA*<br>*P, FAM-TGCTTGCTGCAAAGTATT-MGB*
Camplobacter coli	*GlyA*	*F, AAACCAAAGCTTATCGTGTGC*<br>*R, AGTGCAGCAATGTGTGCAAT*<br>*P, FAM-TAAGCTCCAACTTCATCCG-MGB*
LT-ETEC	*LT*	*F, TTCCCACCGGATCACCAA*<br>*R, CAACCTTGTGGTGCATGATGA*<br>*P, FAM-CTTGGAGAGAAGAACCCT-MGB*
ST-ETEC	*STh*	*F, GCTAAACCAGYAGRGTCTTCAAAA*<br>*R, CCCGGTACARGCAGGATTACAACA*<br>*P, FAM-TGGTCCTGAAAGCATGAA-MGB*
	STp	*F, TGAATCACTTGACTCTTCAAAA*<br>*R, GGCAGGATTACAACAAAGTT*<br>*P, FAM-TGAACAACACATTTTACTGCT-MGB*
STEC	stx1	*F, ACTTCTCGACTGCAAAGACGTATG*<br>*R, ACAAATTATCCCCTGWGCCACTATC*<br>*P, FAM-CTCTGCAATAGGTACTCCA-MGB*
	stx2	*F, CCACATCGGTGTCTGTTATTAACC*<br>*R, GGTCAAAACGCGCCTGATAG*<br>*P, FAM-TTGCTGTGGATATACGAGG-MGB*
Shigella spp.	*ipaH*	*F, CCTTTTCCGCGTTCCTTGA*

medRxiv preprint doi: https://doi.org/10.1101/2024.10.10.24315212; this version posted October 18, 2024. The copyright holder for this preprint (which was not certified by peer review) is the author/funder, who has granted medRxiv a license to display the preprint in perpetuity.
It is made available under a CC-BY 4.0 International license .

		R, CGGAATCCGGAGGTATTGC<br>*P, VIC-CGCCTTTCCGATACCGTCTCTGCA-MGB*
Salmonella enterica	*ttr*	*F, CTCACCAGGAGATTACAACATGG*<br>*R, AGCTCAGACCAAAAGTGACCATC*<br>*P, FAM-CACCGACGGCGAGACCGACTTT-MGB*
Salmonella enterica serovar Typhi	*tviB*	*F, TGTGGTAAAGGAACTCGGTAAA*<br>*R, GACTTCCGATACCGGGATAATG*<br>*P, VIC-TGGATGCCGAAGAGGTAAGACGAGA-MGB*
	sty0201	*F, CGCGAAGTCAGAGTCGACATAG*<br>*R, AAGACCTCAACGCCGATCAC*<br>*P, FAM-CAGCCTGCTCCAGAACA-MGB*
Cryptosporidium hominus	*LIB13*	*F, TCCTTGAAATGAATATTTGTGACTCG*<br>*R, AAATGTGGTAGTTGCGGTTGAAA*<br>*P, FAM-CTTACTTCGTGGCGGCGT-MGB*
Cryptosporidium parvum	*LIB13*	*F, TCCTTGAAATGAATATTTGTGACTCG*<br>*R, TTAATGTGGTAGTTGCGGTTGAAC*<br>*P, FAM-TATCTCTTCGTAGCGGCGTA-MGB*
Cryptosporidium spp.	*18S*	*F, GGGTTGTATTTATTAGATAAAGAACCA*<br>*R, AGGCCAATACCCTACCGTCT*<br>*P, FAM-TGACATATCATTCAAGTTTCTGAC-MGB*
Entamoeba histolytica	*18S*	*F, ATTGTCGTGGCATCCTAACTCA*<br>*R, GCGGACGGCTCATTATAACA*<br>*P, FAM-TCATTGAATGAATTGGCCATTT-MGB*
Giardia spp.	*18S*	*F, GACGGCTCAGGACAACGGTT*<br>*R, TTGCCAGCGGTGTCCG*<br>*P, FAM-CCCGCGGCGGTCCCTGCTAG-MGB*

medRxiv preprint doi: https://doi.org/10.1101/2024.10.10.24315212; this version posted October 18, 2024. The copyright holder for this preprint (which was not certified by peer review) is the author/funder, who has granted medRxiv a license to display the preprint in perpetuity.
It is made available under a CC-BY 4.0 International license .

Supplemental Table 3: Infection prevalence for 14 enteric pathogens measured by Luminex xTAG Gastrointestinal Panel (GPP) and TaqMan Array Card (TAC) assays. Stool samples were tested from children at ages 6, 12, and 18 months old in Esmeraldas Province, Ecuador, 2022-2023. Created with script: https://osf.io/4dteq .

GPP Target	N	GPP pos	GPP Prev (95% CI)	TAC pos	TAC Prev (95% CI)
**Viruses**					
Adenovirus_40_41	154	14	9.1 (5.1, 14.8)	13	8.4 (4.6, 14.0)
Norovirus_GI	154	4	2.6 (0.7, 6.5)	7	4.5 (1.8, 9.1)
Norovirus_GII	154	10	6.5 (3.2, 11.6)	15	9.7 (5.6, 15.6)
Rotavirus_A	154	1	0.6 (0.0, 3.6)	12	7.8 (4.1, 13.2)
**Bacteria**					
Campylobacter	154	30	19.5 (13.5, 26.6)	44	28.6 (21.6, 36.4)
ETEC_LT	154	58	37.7 (30.0, 45.8)	57	37.0 (29.4, 45.2)
ETEC_ST	154	9	5.8 (2.7, 10.8)	23	14.9 (9.7, 21.6)
STEC_stx1	154	19	12.3 (7.6, 18.6)	15	9.7 (5.6, 15.6)
STEC_stx2	154	11	7.1 (3.6, 12.4)	6	3.9 (1.4, 8.3)
Shigella	154	25	16.2 (10.8, 23.0)	19	12.3 (7.6, 18.6)
Salmonella	154	124	80.5 (73.4, 86.5)	12	7.8 (4.1, 13.2)
**Protozoa**					
Cryptosporidium	154	17	11.0 (6.6, 17.1)	13	8.4 (4.6, 14.0)
Entamoeba_histolytica	154	2	1.3 (0.2, 4.6)	1	0.6 (0.0, 3.6)
Giardia	154	24	15.6 (10.2, 22.3)	31	20.1 (14.1, 27.3)

medRxiv preprint doi: https://doi.org/10.1101/2024.10.10.24315212; this version posted October 18, 2024. The copyright holder for this preprint (which was not certified by peer review) is the author/funder, who has granted medRxiv a license to display the preprint in perpetuity.
It is made available under a CC-BY 4.0 International license .

Supplemental Figure 1: Median fluorescence intensity (MFI) values for pathogen targets detected by the Luminex GPP Assay. Results are categorized according to TaqMan Array Card (TAC) and Luminex xTAG Gastrointestinal Pathogen Panel (GPP) assay sample results, for positive (+) and negative (–) detection for each target. Created with script: https://osf.io/hfv5r .

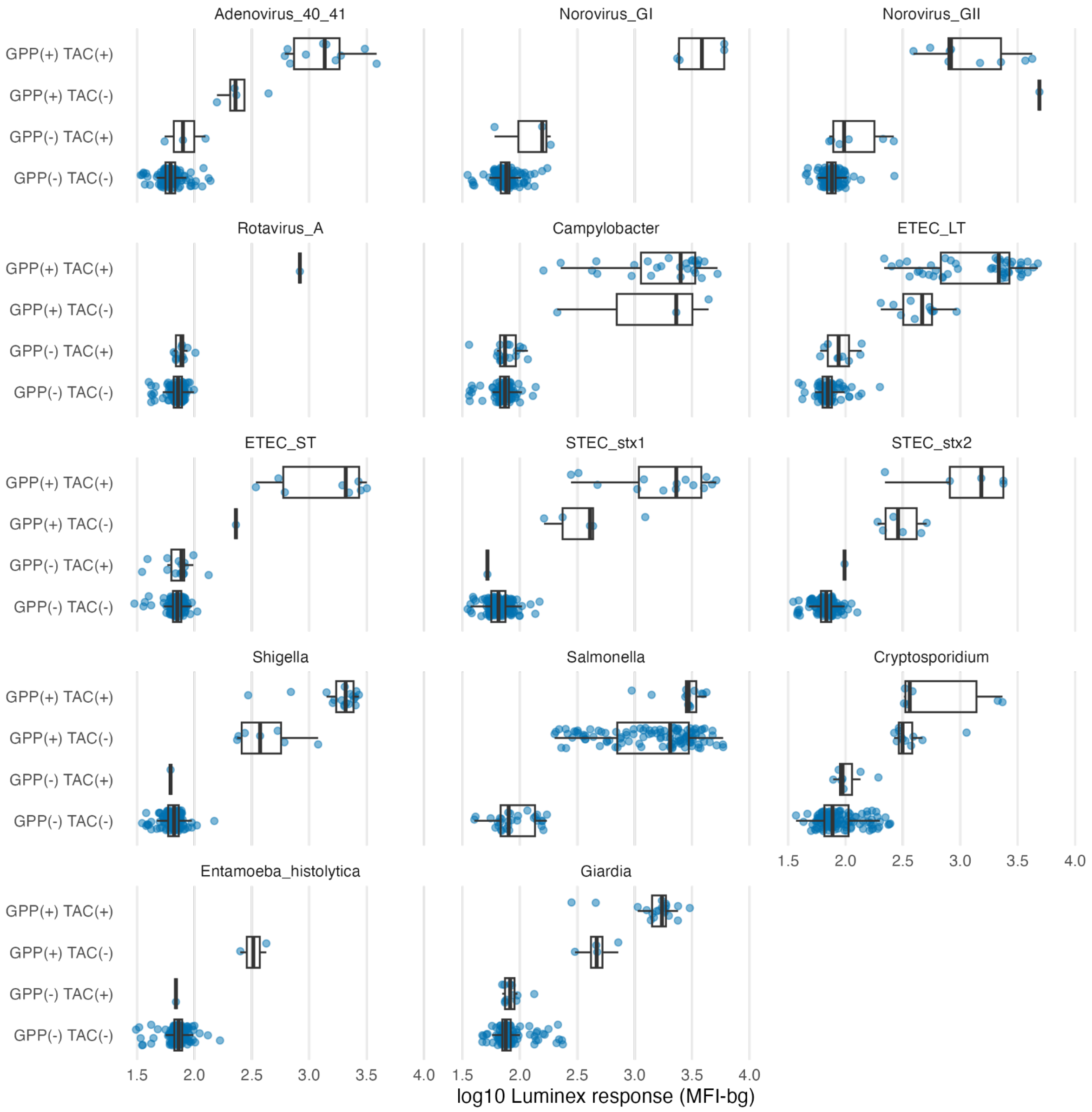

medRxiv preprint doi: https://doi.org/10.1101/2024.10.10.24315212; this version posted October 18, 2024. The copyright holder for this preprint (which was not certified by peer review) is the author/funder, who has granted medRxiv a license to display the preprint in perpetuity.
It is made available under a CC-BY 4.0 International license .

Supplemental Figure 2: Cycle threshold (Ct) values for pathogen associated gene targets detected by the TAC Assay. Multiple gene targets were used for some enteric pathogens in the TaqMan Array Card (TAC) panel. In each comparison, the top row label identifies the Luminex xTAG Gastrointestinal Pathogen Panel (GPP) target, and the second row identifies the TAC target. Results are categorized according to TAC and GPP assay sample results, for positive (+) and negative (–) detection for each GPP target. Created with script: https://osf.io/hfv5r .

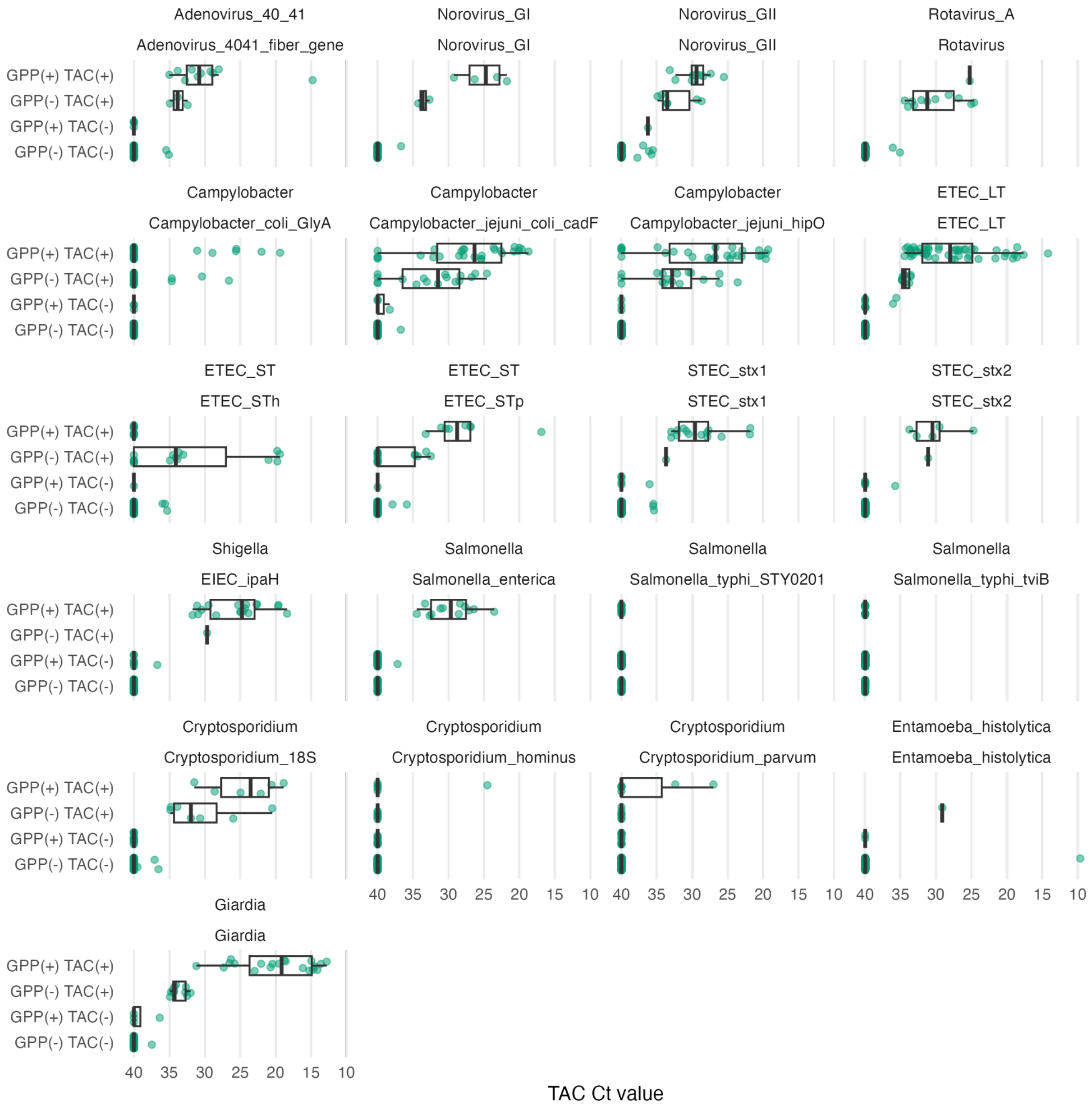

Supplement: 1 [file NIHPP2024.10.10.24315212V2-supplement-1.pdf]
