# Supplementary material for: Multiplex PCR detection of enteric pathogens in a community-based birth cohort in Ecuador: comparison of xTAG-GPP and TaqMan array card assays
Source: medRxiv. 2024 Oct 11:2024.10.10.24315212. Preprint. [Version 1] doi: 10.1101/2024.10.10.24315212 (PMC11482970; doi:10.1101/2024.10.10.24315212)

medRxiv preprint doi: https://doi.org/10.1101/2024.10.10.24315212; this version posted October 11, 2024. The copyright holder for this preprint
(which was not certified by peer review) is the author/funder, who has granted medRxiv a license to display the preprint in perpetuity.
It is made available under a CC-BY 4.0 International license .

Supplemental Table 1: Luminex Gastrointestinal Pathogen Panel (GPP) assay targets and corresponding median fluorescence intensity (MFI) thresholds for positivity

**Analyte**	**Threshold for positivity (MFI)**
Adenovirus *40/41*	≥ 150
Camplobacter	≥ 150
C. difficile Probe-1	≥ 150
C. difficile Probe-2	≥ 150
Cryptosporidium	≥ 250
E. coli O157	≥ 150
E. histolytica	≥ 250
ETEC probe-1	≥ 200
ETEC probe-2	≥ 200
Giardia	≥ 250
Norovirus Probe-1	≥ 200
Norovirus Probe-2	≥ 350
Rotavirus A	≥ 150
Salmonella Probe-1	≥ 100,000 (POS), < 300 (NEG)
Salmonella Probe-2	≥ 200
STEC Probe-1	≥ 150
STEC Probe-2	≥ 150
Shigella	≥ 150
V. cholerae	≥ 150

medRxiv preprint doi: https://doi.org/10.1101/2024.10.10.24315212; this version posted October 11, 2024. The copyright holder for this preprint (which was not certified by peer review) is the author/funder, who has granted medRxiv a license to display the preprint in perpetuity.
It is made available under a CC-BY 4.0 International license .

Supplemental Table 2: TaqMan Array Card (TAC) assay gene targets and corresponding forward (F) and reverse (R) primers and probe (P) sequences.

Organism	Gene Target	Sequence
Adenovirus (40/41)	*Fiber gene*	*F, AACTTTCTCTCTTAATAGACGCC*<br>*R, AGGGGGCTAGAAAACAAAA*<br>*P, FAM-CTGACACGGGCACTCT-MGB*
Norovirus GI	*ORF1-ORF2*	*F, CGYTGGATGCGNTTYCATGA*<br>*R, CTTAGACGCCATCATCATTYAC*<br>*P, FAM-TGGACAGGAGATCGC-MGB*
Norovirus GII	*ORF1-ORF2*	*F, CARGARBCNATGTTYAGR TGGATGAG*<br>*R, TCGACGCCATCTTCATTCACA*<br>*P, FAM-TGGGAGGGCGATCGCAATCT-MGB*
Rotavirus	*NSP3*	*F, ACCATCTWCACRTRACCCTCTATGAG*<br>*R, GGTCACATAACGCCCCTATAGC*<br>*P, FAM-AGTTAAAAGCTAACACTGTCAAA-MGB*
Camplobacter jejuni	*cadF*	*F, CWGCTAAACCATARAAATAAAATTTCTCAC*<br>*R, YTTTGAAGGTAATTTAGATATGGATAATCG*<br>*P, VIC-CATTTTGAYGATTTTTGGCTTGA-MGB*
	hipO	*F, CTTGCGGTCATGATGGACATAC*<br>*R, AGCACCACCCAAACCCTCTTCA*<br>*P, FAM-TGCTTGCTGCAAAGTATT-MGB*
Camplobacter coli	*GlyA*	*F, AAACCAAAGCTTATCGTGTGC*<br>*R, AGTGCAGCAATGTGTGCAAT*<br>*P, FAM-TAAGCTCCAACTTCATCCG-MGB*
LT-ETEC	*LT*	*F, TTCCCACCGGATCACCAA*<br>*R, CAACCTTGTGGTGCATGATGA*<br>*P, FAM-CTTGGAGAGAAGAACCCT-MGB*
ST-ETEC	*STh*	*F, GCTAAACCAGYAGRGTCTTCAAAA*<br>*R, CCCGGTACARGCAGGATTACAACA*<br>*P, FAM-TGGTCCTGAAAGCATGAA-MGB*
	STp	*F, TGAATCACTTGACTCTTCAAAA*<br>*R, GGCAGGATTACAACAAAGTT*<br>*P, FAM-TGAACAACACATTTTACTGCT-MGB*
STEC	stx1	*F, ACTTCTCGACTGCAAAGACGTATG*<br>*R, ACAAATTATCCCCTGWGCCACTATC*<br>*P, FAM-CTCTGCAATAGGTACTCCA-MGB*
	stx2	*F, CCACATCGGTGTCTGTTATTAACC*<br>*R, GGTCAAAACGCGCCTGATAG*<br>*P, FAM-TTGCTGTGGATATACGAGG-MGB*
Shigella spp.	*ipaH*	*F, CCTTTTCCGCGTTCCTTGA*

medRxiv preprint doi: https://doi.org/10.1101/2024.10.10.24315212; this version posted October 11, 2024. The copyright holder for this preprint
(which was not certified by peer review) is the author/funder, who has granted medRxiv a license to display the preprint in perpetuity.
It is made available under a CC-BY 4.0 International license .

		R, CGGAATCCGGAGGTATTGC<br>*P, VIC-CGCCTTTCCGATACCGTCTCTGCA-MGB*
Salmonella enterica	*ttr*	*F, CTCACCAGGAGATTACAACATGG*<br>*R, AGCTCAGACCAAAAGTGACCATC*<br>*P, FAM-CACCGACGGCGAGACCGACTTT-MGB*
Salmonella enterica serovar Typhi	*tviB*	*F, TGTGGTAAAGGAACTCGGTAAA*<br>*R, GACTTCCGATACCGGGATAATG*<br>*P, VIC-TGGATGCCGAAGAGGTAAGACGAGA-MGB*
	sty0201	*F, CGCGAAGTCAGAGTCGACATAG*<br>*R, AAGACCTCAACGCCGATCAC*<br>*P, FAM-CAGCCTGCTCCAGAACA-MGB*
Cryptosporidium hominus	*LIB13*	*F, TCCTTGAAATGAATATTTGTGACTCG*<br>*R, AAATGTGGTAGTTGCGGTTGAAA*<br>*P, FAM-CTTACTTCGTGGCGGCGT-MGB*
Cryptosporidium parvum	*LIB13*	*F, TCCTTGAAATGAATATTTGTGACTCG*<br>*R, TTAATGTGGTAGTTGCGGTTGAAC*<br>*P, FAM-TATCTCTTCGTAGCGGCGTA-MGB*
Cryptosporidium spp.	*18S*	*F, GGGTTGTATTTATTAGATAAAGAACCA*<br>*R, AGGCCAATACCCTACCGTCT*<br>*P, FAM-TGACATATCATTCAAGTTTCTGAC-MGB*
Entamoeba histolytica	*18S*	*F, ATTGTCGTGGCATCCTAACTCA*<br>*R, GCGGACGGCTCATTATAACA*<br>*P, FAM-TCATTGAATGAATTGGCCATTT-MGB*
Giardia spp.	*18S*	*F, GACGGCTCAGGACAACGGTT*<br>*R, TTGCCAGCGGTGTCCG*<br>*P, FAM-CCCGCGGCGGTCCCTGCTAG-MGB*

medRxiv preprint doi: https://doi.org/10.1101/2024.10.10.24315212; this version posted October 11, 2024. The copyright holder for this preprint (which was not certified by peer review) is the author/funder, who has granted medRxiv a license to display the preprint in perpetuity.
It is made available under a CC-BY 4.0 International license .

Supplemental Table 3: Summary of multiplex PCR test results for Luminex xTAG Gastrointestinal Pathogen Panel (GPP) and TaqMan Array Card (TAC). Test results from 154 samples measured among children at ages 6, 12, and 18 months old in Esmeraldas Province, Ecuador, 2022-2023. Test results are summarized by whether they were positive (+) or negative (–) by GPP and TAC. Methods include details on estimation of agreement, Cohen's kKappa, and McNemar's test for differences between assays. Created with script: https://osf.io/4dteq .

Assay Target	GPP - TAC -	GPP + TAC -	GPP - TAC +	GPP + TAC +	GPP Prevalence % (95% Confidence Interval)	TAC Prevalence % (95% Confidence Interval)	% Agreement (95% Confidence Interval)	Kappa Agreement (95% Confidence Interval)	McNemar's p-value
Adenovirus (40/41)	137	4	3	10	9.1 (5.1, 14.8)	8.4 (4.6, 14)	95.5 (90.9, 98.2)	0.72 (0.56, 0.9)	0.71
Norovirus GI	147	0	3	4	2.6 (0.7, 6.5)	4.5 (1.8, 9.1)	98.1 (94.4, 99.6)	0.72 (0.57, 0.9)	0.08
Norovirus GII	138	1	6	9	6.5 (3.2, 11.6)	9.7 (5.6, 15.6)	95.5 (90.9, 98.2)	0.70 (0.54, 0.9)	0.06
Rotavirus	142	0	11	1	0.6 (0, 3.6)	7.8 (4.1, 13.2)	92.9 (87.6, 96.4)	0.14 (0.06, 0.2)	0.00
Campylobacter spp.	107	3	17	27	19.5 (13.5, 26.6)	28.6 (21.6, 36.4)	87.0 (80.7, 91.9)	0.65 (0.50, 0.8)	0.00
LT-ETEC	87	10	9	48	37.7 (30, 45.8)	37 (29.4, 45.2)	87.7 (81.4, 92.4)	0.74 (0.58, 0.9)	0.82
ST-ETEC	NA	1	15	8	5.8 (2.7, 10.8)	14.9 (9.7, 21.6)	89.6 (83.7, 93.9)	0.45 (0.32, 0.6)	0.00
STEC stx1	134	5	1	14	12.3 (7.6, 18.6)	9.7 (5.6, 15.6)	96.1 (91.7, 98.6)	0.80 (0.65, 1.0)	0.10
STEC stx2	142	6	1	5	7.1 (3.6, 12.4)	3.9 (1.4, 8.3)	95.5 (90.9, 98.2)	0.57 (0.42, 0.7)	0.06
Shigella spp.	128	7	1	18	16.2 (10.8, 23)	12.3 (7.6, 18.6)	94.8 (90.0, 97.7)	0.79 (0.63, 0.9)	0.03
Salmonella spp.	30	112	0	12	80.5 (73.4, 86.5)	7.8 (4.1, 13.2)	27.3 (20.4, 35.0)	0.04 (-0.00, 0.1)	0.00
Cryptosporidium spp.	130	11	7	6	11 (6.6, 17.1)	8.4 (4.6, 14)	88.3 (82.2, 92.9)	0.34 (0.18, 0.5)	0.35
Entamoeba histolytica	151	2	1	0	1.3 (0.2, 4.6)	0.6 (0, 3.6)	98.1 (94.4, 99.6)	-0.01 (-0.16, 0.1)	0.56
Giardia spp.	119	4	11	20	15.6 (10.2, 22.3)	20.1 (14.1, 27.3)	90.3 (84.4, 94.4)	0.67 (0.51, 0.8)	0.07

medRxiv preprint doi: https://doi.org/10.1101/2024.10.10.24315212; this version posted October 11, 2024. The copyright holder for this preprint (which was not certified by peer review) is the author/funder, who has granted medRxiv a license to display the preprint in perpetuity.
It is made available under a CC-BY 4.0 International license .

Supplemental Figure 1: Median fluorescence intensity (MFI) values for pathogen targets detected by the Luminex GPP Assay. Results are categorized according to TaqMan Array Card (TAC) and Luminex xTAG Gastrointestinal Pathogen Panel (GPP) assay sample results, for positive (+) and negative (–) detection for each target. Created with script: https://osf.io/hfv5r .

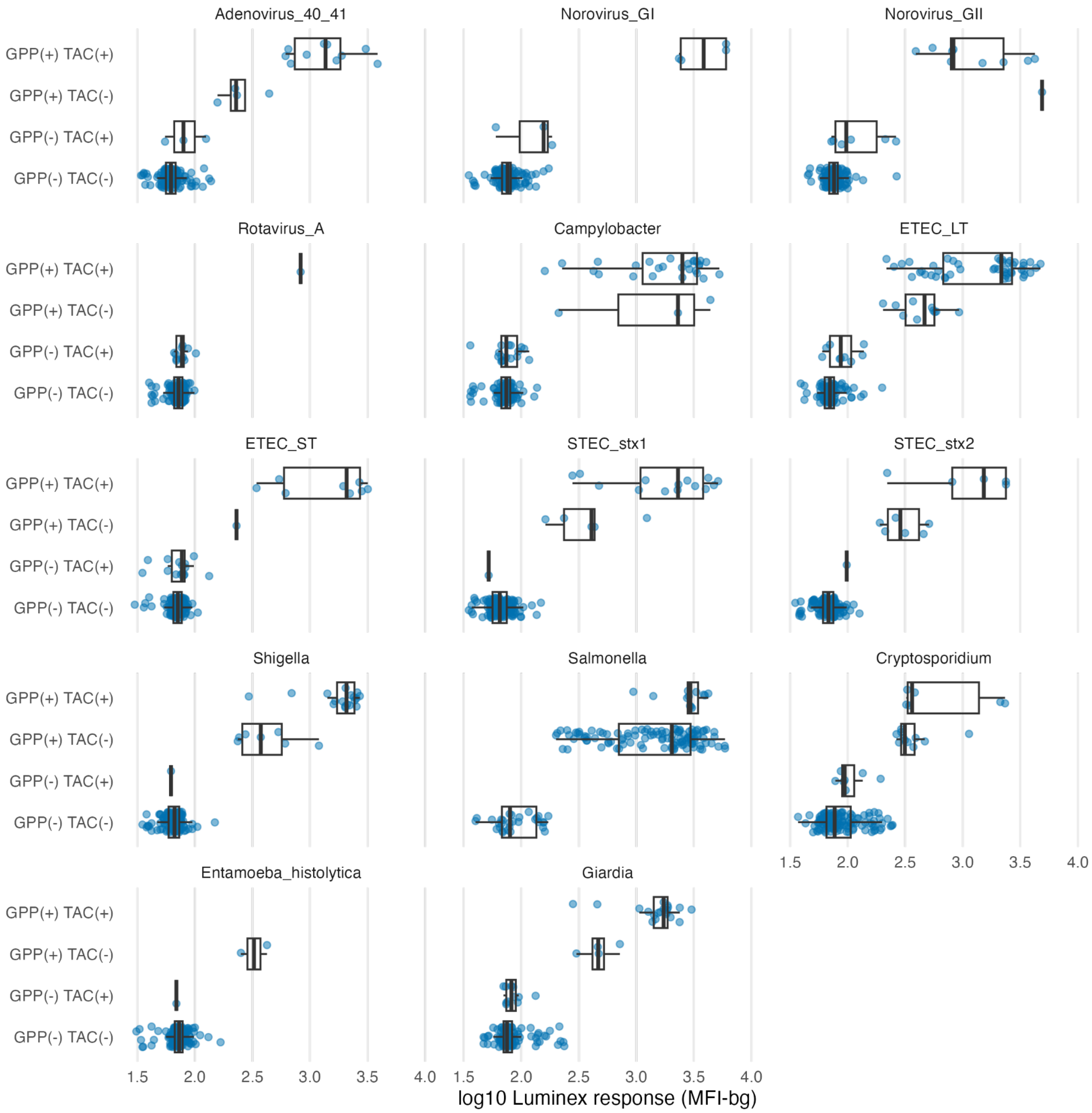

medRxiv preprint doi: https://doi.org/10.1101/2024.10.10.24315212; this version posted October 11, 2024. The copyright holder for this preprint (which was not certified by peer review) is the author/funder, who has granted medRxiv a license to display the preprint in perpetuity.
It is made available under a CC-BY 4.0 International license .

Supplemental Figure 2: Cycle threshold (Ct) values for pathogen associated gene targets detected by the TAC Assay. Multiple gene targets were used for some enteric pathogens in the TaqMan Array Card (TAC) panel. In each comparison, the top row label identifies the Luminex xTAG Gastrointestinal Pathogen Panel (GPP) target, and the second row identifies the TAC target. Results are categorized according to TAC and GPP assay sample results, for positive (+) and negative (–) detection for each GPP target. Created with script: https://osf.io/hfv5r .

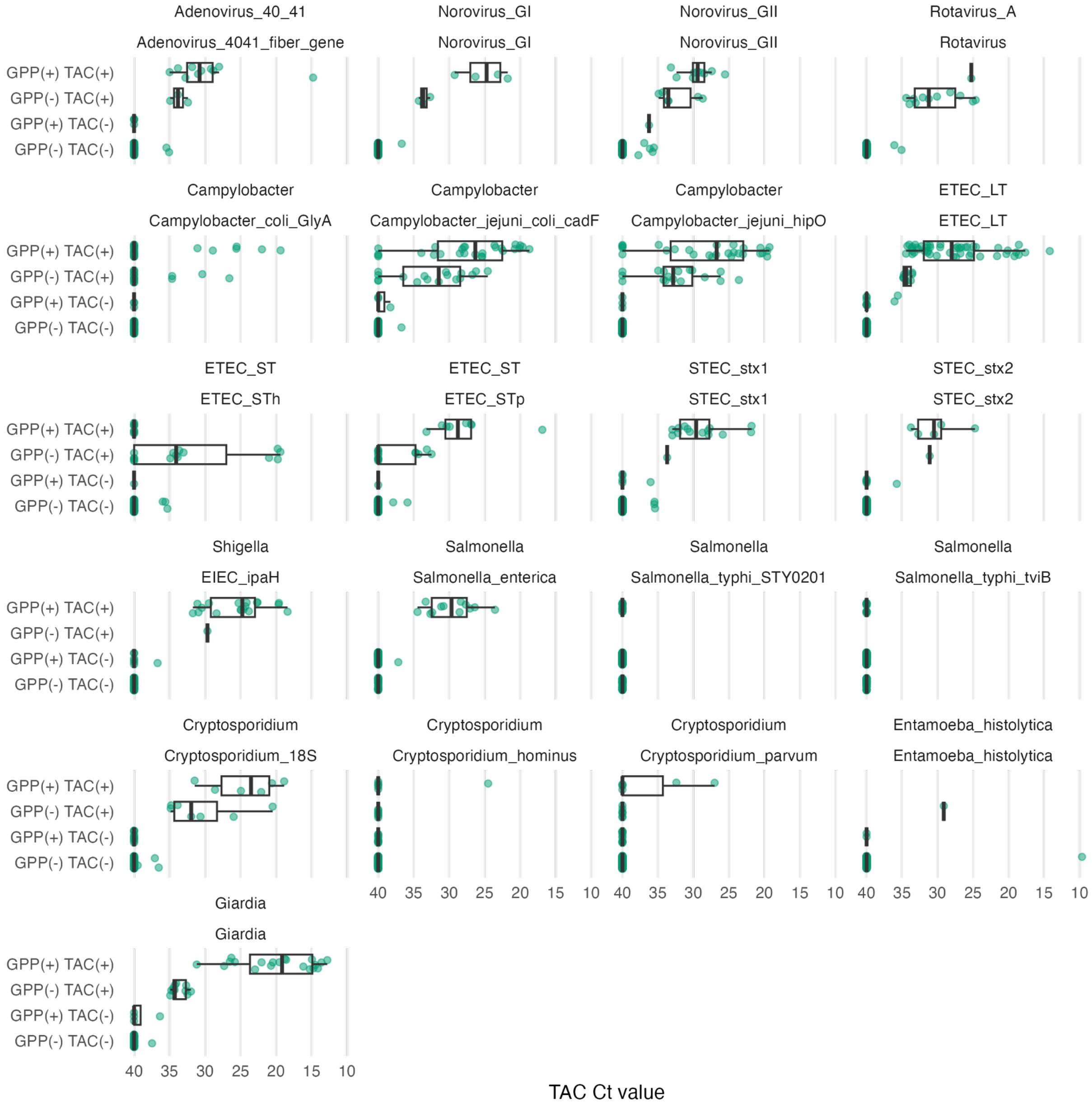

Supplement: Supplementary file 1 [file NIHPP2024.10.10.24315212V1-supplement-1.pdf]
